# Tuning Pluronic Hydrogel Properties via Ionic Strength and Hyaluronic Acid for Optimized Rheology and Drug Delivery Performances

**DOI:** 10.1021/acsomega.5c08070

**Published:** 2025-12-04

**Authors:** Anderson Ferreira Sepulveda, Lucas Ferreira de Oliveira, Agatha Maria Pelosine, Wendel A. Alves, Daniele Ribeiro de Araujo

**Affiliations:** † Center for Natural and Human Sciences, 74362 Federal University of ABC, 09280-560 Santo André, SP, Brazil; ‡ Department of Biophysics, Federal University of São Paulo, 04044020 São Paulo, SP, Brazil

## Abstract

New formulations based on polymeric matrices have demonstrated promising results as drug delivery systems for intra-articular administration. In this study, we propose a five-step approach to guide the selection of the most adequate formulation, considering some features, such as sol–gel transition temperature, mechanical strength, system stability, and drug release performance. As a model, we developed hydrogels composed of Pluronic F68, F108, and F127 at 20% (w/v), combined with hyaluronic acid (HA) of 10, 200, and 1500 kDa at 1% (w/v), along with NaCl (154 mM) and MgCl_2_ (0.9 mM), to simulate the intra-articular environment. Physicochemical characterization was conducted through rheological analysis, texture profile analysis, dynamic light scattering, and drug release studies. The addition of salts or HA of varying molecular masses influenced the organization of the hydrogel, promoting either greater structural ordering (as observed with MgCl_2_ and 200 kDa HA) or reduced structuring (as with 1500 kDa HA). Notably, more rigid systems may impair injectability and alter drug release profiles. The incorporation of different HA molecular masses disrupted dense micellar packing—particularly in F127-based hydrogels—affecting the gel network while promoting either elastic or entangled characteristics. Furthermore, drug release was slowed in systems where both HA and Pluronic formed an entangled network. Overall, the evaluation of these drug carrier systems allowed the identification of PL formulations with optimal mechanical, structural, and release properties, offering improved efficiency for intra-articular drug delivery applications.

## Graphical abstract

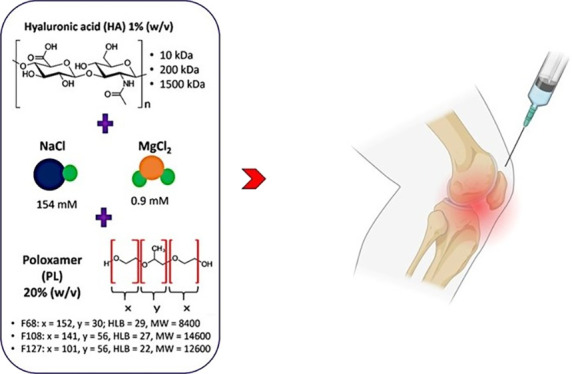

## 1 Introduction

Osteoarthritis and rheumatoid arthritis are the main inflammatory conditions associated with discomfort, stiffness, and decreased joint function. The causes may be related to the degeneration of connective tissues due to mechanical damage or irregular metabolism, inflammatory process, and immune system malfunction associated with the aging process.^1^

Several drug carrier systems have been proposed as new materials capable of controlling the release of drugs or bioactives by the intra-articular route, such as hydrogels, emulsions, and micro- and nanoparticles, based on synthetic and natural polymers, lipids, and other materials. Synthetic polymer hydrogels have shown success in intra-articular applications, mainly those composed of poloxamers or Pluronic (PL).^2,3^ However, a more in-depth investigation of the PL structural arrangement and physicochemical behavior is still needed, especially in the presence of other polymers such as hyaluronic acid (HA). Furthermore, studies have shown that the presence of salts in formulations can affect the material’s structural organization, affecting the formulation’s viscosity and its potential application by the intra-articular route.

PL are copolymers composed of hydrophilic poly­(ethylene oxide) (PEO) and hydrophobic poly­(propylene oxide) (PPO) blocks, arranged as PEO_*x*_-PPO_*y*_-PEO_*x*_ (Figure 1). The *x* and *y* values confer different physicochemical properties, making PL capable of interacting with hydrophilic and hydrophobic interfaces. In an aqueous solution, at an appropriate temperature, and when the concentrations of these copolymers are above the critical micelle concentration (CMC), the blocks self-organize into micelles.^4–6^ PEO and PPO units can interact with water, and with increasing temperature, the hydrophobic units of PPO are dehydrated and aggregate while the hydrophilic PEO units remain hydrated. Subsequently, at high PL concentrations, micelles self-organize (“packing”) into cubic, hexagonal, and lamellar phases, producing partially rigid structures with high viscosity, characterized by a sol–gel transition temperature (*T*_sol–gel_), where these materials remain fluid below while they are semisolid hydrogels above the *T*_sol–gel_.^6–8^

**Figure 1:**
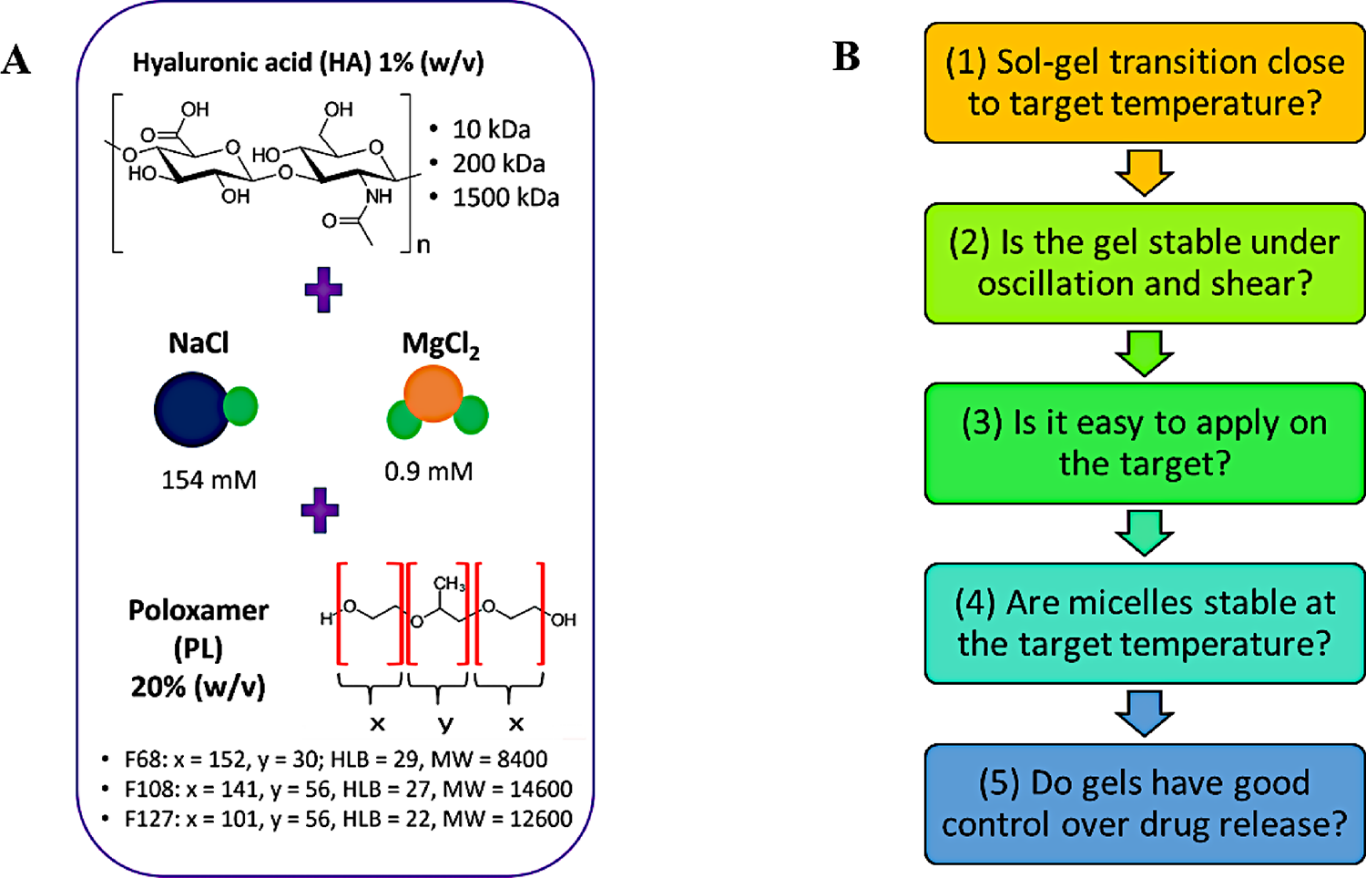
(A) Formulations based on Pluronics F68, F108, and F127 (20% w/v in ultrapure water) were combined with 154 mM NaCl or 0.9 mM MgCl_2_ and 1% (w/v) of 10 kDa, 200 kDa, or 1500 kDa hyaluronic acid (HA). (B) In this work, we propose five steps to optimize the development and characterization of new materials for pharmacological or cosmetic use.

PL F127 (PEO_98_-PPO_67_-PEO_98_) is one of the most studied PL, presenting an average molar mass of 12,600 g/mol, a hydrophilic–lipophilic balance of 22, and a CMC of 2.8 μM. As systems for local anesthetics delivery, the drug model in this study, F127, has been the basis of several studies. Initial work by Paavola and collaborators showed that the use of lidocaine and ketoprofen incorporated into F127 hydrogels (20–30% copolymer containing liposomes) increased the permeation of these drugs through the isolated porcine dural membrane.^7^ Subsequently, new studies addressed the preparation and characterization processes of F127 hydrogels containing diclofenac and lidocaine,^8^ lidocaine,^9,10^ ropivacaine,^11^ dibucaine and tetracaine,^12^ and bupivacaine.^3^

Due to its high viscosity and natural presence in joints, hyaluronic acid (HA) has been used in association with PL hydrogels. HA is a linear polysaccharide composed of alternating (1→4)-β linked d-glucuronic and (1→3)-β linked *N*-acetyl-d-glucosamine residues and is a member of the glycosaminoglycans (GAGs) (Figure 1), forming a natural component of the extracellular medium, especially important in cartilaginous and connective tissues, with mucoadhesive and biodegradable properties.^13,14^ The presence of OH groups confers it a strong polar characteristic, while its high molar mass determines unique viscoelastic and rheological properties.^14,15^ HA plays an essential role in avoiding friction between bones, lubricating joints, and keeping connective tissues intact due to its high viscosity.^15^ In particular, the HA molecular mass can play different roles since large chains are space-filling, antiangiogenic, and immunosuppressive and stimulate fibroblast proliferation,^16^ while the HA intermediate molecular mass is proinflammatory, immunostimulatory, and highly angiogenic. Additionally, smaller oligosaccharides are antiapoptotic and induce heat shock proteins. Then, variably sized fragments can trigger different signal transduction pathways.^14^

Recent advances in the rheological characterization of soft materials have been strongly influenced by studies that connect molecular organization, viscoelastic behavior, and processing performance.^3,5^ The work published by Lu et al. provides a contribution by correlating supramolecular interactions with the macroscopic rheological response of complex systems. This molecular-level understanding helps clarify how cross-linking dynamics and noncovalent interactions govern the elasticity, flow, and self-healing properties of structured materials.^17^ Complementarily, the review by Calafel et al. establishes rheology as a central tool for evaluating the printability and mechanical integrity of hydrogel-based inks in additive manufacturing. By linking parameters such as yield stress, shear-thinning behavior, and thixotropy to extrusion performance, it defines a “rheological window” that guides the formulation of materials with optimized flow and shape fidelity during bioprinting.^18^ Meanwhile, the comprehensive overview published by Liu and Chen expands the scope of rheological analysis toward biomedical applications, particularly in drug delivery. This work highlights how viscoelasticity influences key functional aspects such as injectability, swelling, and controlled release, emphasizing that rheological properties are not merely descriptive but predictive of therapeutic performance.^19^ Together, these studies demonstrate how rheology bridges fundamental chemistry and practical applications, serving as a unifying parameter to design, process, and evaluate advanced soft materials for biotechnological and pharmaceutical use.

Herein, hydrogels based on F127, F108, and F68, either isolated or combined with HA of 10, 200, or 1500 kDa, were prepared in the presence of the salts NaCl and MgCl_2_ at physiological concentrations, carrying the local anesthetic lidocaine hydrochloride. The formulation’s design was based on both the hydrophilic–lipophilic balance (HLB) value and the propylene oxide (PO) and ethylene oxide (EO) unit ratio. Considering the search for intr-articular application, we propose a series of steps to find the best hydrogel performance, evaluating the influence of the HA molecular mass and ionic environmental force (Figure 1B): (1) the gel must undergo a sol–gel transition close to physiological temperature (∼37 °C), (2) the gel should present stability under the oscillation and shear typical of joint movement, (3) gels must not exhibit high viscosity that makes it challenging to apply the material by syringe (syringability), (4) micelles must display structural stability at physiological temperature, and (5) the gels must show reasonable control over drug release over time. In this sense, hydrogels were then characterized by their mechanical properties to analyze stability under varying physical conditions and dynamic light scattering (DLS) to measure the hydrodynamic diameter of the vesicles in an aqueous medium. Release tests were also carried out to verify the effectiveness of controlling drug release.

## 2 Materials and Methods

### 2.1 Excipients and Drug

Pluronic F127 (F127), Pluronic F108 (F108), and Pluronic F68 (F68) (Table 1) were purchased from Sigma-Aldrich, Chem. Corp., Saint Louis, MO, USA. Hyaluronic acid (HA; 98% purity; 10, 200, or 1500 kDa) was a gift from Symbios Especialidades Ltda., São Carlos, SP, Brazil. They were used as excipients. Lidocaine hydrochloride (LDC; pharmaceutical grade) was kindly received from Cristália Produtos Químicos Farmacêuticos Ltda. (Itapira, São Paulo, Brazil).

**Table 1:** Some Physicochemical Properties of Poloxamers F127, F108, and F68^a^

	*n*_EO_	*n*_PO_	*n*_EO_:*n*_PO_	HLB	MW	CMC (37 °C)
F127	101	56	2.8:1	22	12,600	0.01 mM
F108	141	56	2.5:1	27	14,600	0.07 mM
F68	152	30	5.1:1	29	8400	1.5 mM

a *n*_EO_ and *n*_PO_ are the number of ethylene oxide (EO) and propylene oxide (PO) monomers, respectively. *n*_EO_:*n*_PO_ is the chain’s ratio between EO and PO monomers. HLB is hydrophilic–lipophilic balance. MW is the molecular weight of each poloxamer. CMC is the critical micellar concentration, at 37 °C.

### 2.2 Hydrogel Preparation

F127, F108, and F68 solutions (Table 1) were prepared in ultrapure water (18.2 MΩ), with a concentration of 20% (w/v), and kept under constant stirring (500 rpm), at ∼4 °C, until complete dissolution. Meanwhile, it was incorporated with 1% (w/v) HA (10, 200, or 1500 kDa) and NaCl (154 mM) or MgCl_2_ (0.91 mM), in physiological concentrations.^20^ For clarification, all formulation compositions were organized by salt type: group NaCl with F68/NaCl, F108/NaCl, and F127/NaCl isolated or in association with HA (10, 200, or 1500 kDa) and group MgCl_2_ with F68/MgCl_2_, F108/MgCl_2_, and F127/MgCl_2_ isolated or in association with HA (10, 200, or 1500 kDa). After formulation selection, 2% LDC (w/v, 20 mg/mL) was incorporated as a drug model for release tests.

### 2.3 Hydrodynamic Diameter and Polydispersity

The dynamic light scattering technique determines the micelle hydrodynamic diameter and size distribution (polydispersity). A NanoSeries ZS90 particle analyzer from Malvern Instruments was used. Measurements were carried out at a concentration of 5% (for F127 samples) and 10% (F68 samples), considering the critical micellar concentration (Table 1), and were prepared at least 24 h in advance to promote stability. The samples were evaluated to determine the average hydrodynamic diameter and polydispersity value.

### 2.4 Rheological and Injectability Characterization

Rheological characterization enabled the analysis of the stability and mechanical properties of viscoelastic materials under various physical conditions. Temperature (temperature sweep), frequency (frequency sweep), and shear (flow curve and amplitude sweep) tests were performed as follows:

Temperature sweep: The elastic modulus *G*′, viscous modulus *G*″, and viscosity η were analyzed in the temperature range between 10 and 60 °C, with a frequency of 1 Hz and a shear stress of 1 Pa.

Frequency sweep: the elastic modulus *G*′ and viscous modulus *G*″ were analyzed at a low frequency between 0.1 and 10 Hz, with a shear strain of 1%, at 25 °C.

Flow curve: the shear stress σ and the viscosity η were measured in a flow rate (γ̇) from 0 to 100 s^–1^, at 25 °C.

Amplitude sweep: to study the stability of the hydrogels under shearing, *G*′ and *G*″ were measured under variation of shear strain.

All measurements were taken on an oscillatory rheometer (Kinexus Lab Rheometer, Malvern Instruments, United Kingdom) with a plate–plate geometry and a gap of 0.1 mm. GraphPad Prism and Polyana software were used to analyze the results.

Injectability analysis was performed in a texture analyzer (TA.HDplusC, Stable Micro Systems, Surrey, UK), with a 5 kg load cell. All formulations were left at 25 °C for at least 6 h before the experiment. One mL of each formulation was placed in a 1 mL syringe (Descarpack) fitted with 22 G1″ (25 × 0.70) needles. Using 0.05 N as the trigger force, the compression plate above the syringe plunger descended 25 mm at a velocity of 1 mm/s, simulating the typical speed of manual injection.^21^

### 2.5 Release Profiles and Mathematical Modeling

For *in vitro* release assays, a Franz-type vertical diffusion cell system (Microette Plus, Hanson Research Co.) with an area of 1.7 cm^2^ was used. Donor and receptor compartments were separated by an acetate membrane cellulose (with a molecular exclusion pore of 15,000 Da, Spectrapore). The formulations were placed in the donor compartment (1.0 mL), and the receptor was filled with 7 mL of buffer solution (20 mM sodium phosphate buffer with 150 mM NaCl, pH 7.4, 37 °C) under magnetic stirring (100 rpm). At predetermined intervals (15, 30, and 60 min), aliquots were removed and analyzed by spectrophotometry (UV–vis, at 224 nm) for LDC quantification.

To determine the drug release mechanisms from different formulations, the mathematical models of zero-order (eq 1), Higuchi (eq 2), Hixson–Crowell (eq 3), and Korsmeyer–Peppas (eq 4) were applied,^22^ as described by eqs 1–4, respectively. Data were adjusted using the linear regression method, and the coefficient of determination (*R* ^2^) was evaluated.

$$Q_{t}=Q_{0}+K_{0}t$$ 1

where *Q*_*t*_ is the amount of drug released in time *t*, *Q*_0_ is the initial amount of drug in the solution, and *K*_0_ is the zero-order release constant.

$$Q_{t}=K_{H}\times \sqrt{t}$$ 2

where *Q*_*t*_ is the amount of drug released in time *t* and *K*_H_ is the Higuchi release constant.

$${Q_{0}}^{1/3}={Q_{t}}^{1/3}+K_{s}t$$ 3

where *Q*_0_ is the initial amount of drug in the pharmaceutical form, *Q*_*t*_ is the amount of drug remaining in the pharmaceutical form at the end of time *t*, and *K*_s_ is the Hixson–Crowell release constant.

$$\frac{M_{t}}{M}=K_{k}\times t^{n}$$ 4

where *M*_*t*_/*M* < 0.6, where *M*_*t*_/*M* represents the fraction of drug released at the end of time *t*; *K*_k_ is the Korsmeyer–Peppas release constant; *n* is the release exponent, being an indicator of the release mechanism: *n* ≤ 0.5, Fickian transport; *n* = 0.5, classical Fickian diffusion (Higuchi); 0.5 < *n* < 1.0, non-Fickian transport; and *n* = 1, zero-order release kinetics.

### 2.6 Statistical Analysis

All experiments were performed in triplicate, and data are shown as the mean and standard deviation. The statistics were performed using one-way ANOVA with Tukey’s multiple-comparison test.

## 3 Results and Discussion

### 3.1 Step 1: Sol–Gel Transition Temperature (*T*_sol–gel_)

Formulations based on PL tend to provide better control over drug release because they become well-structured and viscoelastic at the target temperature, as observed for systems containing F127, F108, and F68 or their combinations. The structural and rheological properties of PL hydrogels are highly temperature-sensitive, and the incorporation of salts, drugs, or other additives (such as nanocarriers, oils, or polymers) is crucial to understanding possible shifts in values.

In the presence of NaCl, the F68 hydrogel containing 200 kDa HA exhibited the lowest viscosity, reaching up to 0.8 Pa s (Figure 2A) and up to 64 Pa s under another condition (Figure 2D), indicating a more fragile structure compared to those of F108 and F127. Conversely, F108 and F127 hydrogels containing 200 kDa HA displayed significantly higher viscosities, ranging from 1000 to 1200 Pa s (Figure 2B) and 1500–1750 Pa s (Figure 2C), respectively. These differences are primarily attributed to the molecular architecture of the poloxamers: F68 has shorter PPO blocks and a lower hydrophobic content than F108 or F127 (Table 1). Shorter PPO blocks generate smaller micelles with weaker hydrophobic associations, resulting in a less cohesive gel network. In contrast, F108 and particularly F127 form larger, more strongly interacting micelles, producing a denser intermicellar network and, consequently, higher bulk viscosity.

**Figure 2:**
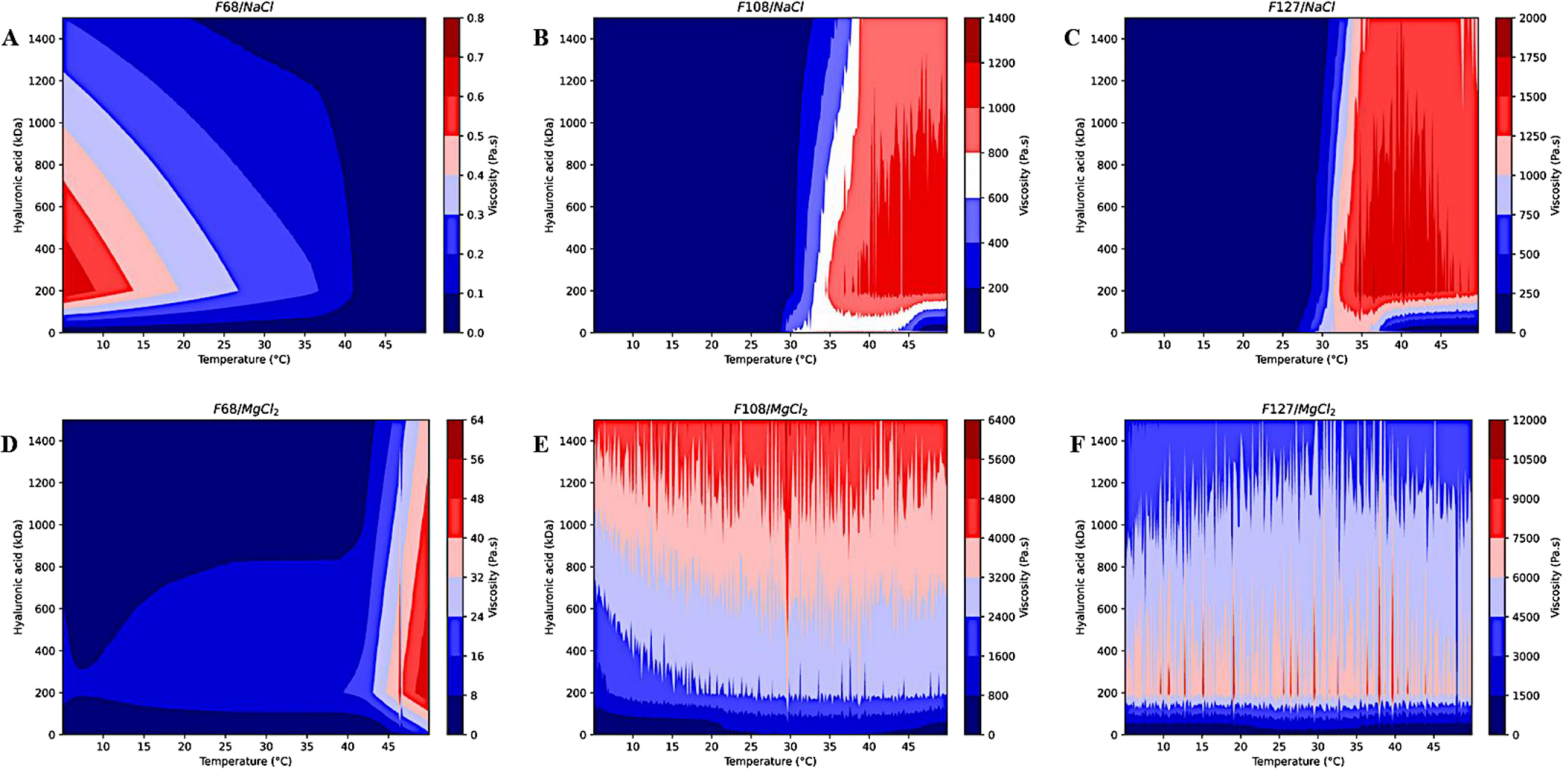
Rheograms obtained from F68, F108, and F127 temperature sweeps containing 154 mM NaCl (A–C) or 0.9 mM MgCl_2_ (D–F) and different-molecular-weight hyaluronic acid (HA). Only F108/NaCl/200 kDa HA (B) and F127/NaCl/200 kDa HA (C) display a sol–gel transition temperature close to the physiological temperature. F68 with NaCl or MgCl_2_, independently of the kind of HA, is in the sol state (A, D). MgCl_2_ increases the viscosity in F108 and F127 formulations (E, F).

Additionally, the high-molecular-weight HA (200 kDa) can interact with and become physically entrapped within the interstitial spaces of strong micellar networks, further increasing viscosity through polymer–micelle entanglement and bridging. In weaker F68 networks, HA chains are less confined, contributing minimally to the viscosity. Moreover, NaCl screens the electrostatic repulsion along HA chains and reduces their hydrodynamic volume, which alters HA partitioning between the aqueous phase and the micellar domains. In the case of F68, the combination of salt-induced HA compaction and competition for water further destabilizes micelle–micelle interactions, resulting in a fragile network and low viscosity. In contrast, the robust micellar networks of F108 and F127 remain cohesive even in the presence of NaCl, allowing HA (even in a compacted form) to maintain or enhance network connectivity and thus sustain higher viscosity values.

The incorporation of MgCl_2_ into the formulations led to a general increase in viscosity compared to that of systems containing NaCl. Moreover, F108/MgCl_2_ and F127/MgCl_2_ hydrogels exhibited stable viscosity profiles over the temperature range and regardless of HA molecular weight (Figure 2E,F). Unlike NaCl-containing systems, MgCl_2_-based formulations showed viscosity values that varied with the HA molecular weight. Specifically, F68/MgCl_2_ with 200 kDa HA showed viscosity lower than those of formulations containing other HA molecular weights but displayed a more rigid structure above 40 °C (Figure 2D). F127/MgCl_2_ also demonstrated markedly higher viscosity in the presence of 200 kDa HA (6000–7500 Pa s; Figure 2F), while F108/MgCl_2_ hydrogels exhibited a viscosity trend of 0 < 10 kDa < 200 kDa < 1500 kDa (Figure 2E).

Several cooperative mechanisms explain the higher viscosity of MgCl_2_-based systems: (1) Mg^2+^, being divalent, can form ionic bridges between carboxylate groups of HA chains, partially cross-linking the network and restricting molecular mobility, which increases both elastic and viscous responses; (2) the higher charge density of Mg^2+^ more effectively screens HA charges than Na^+^, reducing electrostatic repulsion and promoting closer HA–HA interactions; (3) divalent ions such as Mg^+^ also alter micellar hydration, promoting dehydration of the PPO core and strengthening micelle–micelle associations; and (4) MgCl_2_ exhibits a stronger salting-out effect than NaCl, which enhances hydrophobic interactions among PPO blocks and reinforces gel structuring.

The *T*_sol–gel_ transition reflects the hydrogel’s ability to self-organize for controlled release at physiological temperature. In the presence of NaCl, F68 (without HA) and F68 + 10 kDa HA showed *T*_sol–gel_ values above 30 °C, whereas F108 and F127 tended to gel at temperatures ≥ 25 °C, independent of HA molecular weight (Figure S1A). The addition of MgCl_2_ decreased *T*_sol–gel_ for F108 and F127 (to below 25 °C), while F68 formulations transitioned to gel only above 38 °C (Figure S1B). These findings indicate that the contribution of HA to overall gel structuring is relatively minor compared to the dominant influence of the poloxamer type, hydrophobic–hydrophilic balance, and the nature of the salt in determining gel formation and stability near physiological conditions.

For intra-articular applications, the formulation must be structured (in the gel state) at 37 °C to ensure sustained retention and release of the bioactive compound. As shown in Figure 2 and Figure S1, F108 and F127 hydrogels containing NaCl and any HA molecular weight are suitable for intra-articular delivery since they remain in the sol state at storage temperature and undergo sol–gel transition near physiological temperature. However, in the presence of MgCl_2_, all F108 and F127 formulations were already in the gel state at storage temperature, which could hinder injectability. Conversely, F68/MgCl_2_ systems exhibited a sol–gel transition only above physiological temperature, limiting their suitability for controlled drug delivery applications.^23^

### 3.2 Step 2: Gel Stability

As indicated by their decreasing hydrophilic–lipophilic balance (HLB) values (Table 1), the PL studied herein can differentiate along the series by F68 > F108 > F127. Comparatively more hydrophobic PL gels, due to their stronger micellar interactions and tighter network structures, typically exhibit predominant elastic behavior, with *G*′ > *G*″, reflecting a highly structured and elastic gel. Figure S2 illustrates the evolution of *G*″/*G*′ as a function of frequency at 37 °C, showcasing similar dilatant behavior. F68/NaCl appears quite disorganized, displaying a more viscous character (*G*″ > *G*′), particularly in formulations with 200 and 1500 kDa HA (Figure S2A). However, the addition of MgCl_2_ to F68 formulations led to structural modifications, favoring the elastic behavior (*G*″ < *G*′), with the *G*″/*G*′ ratio ranging between 0.4 and 0.45 (Figure S2D), like the curves obtained for F108/NaCl and F127/NaCl (Figure S2B,C).

As observed above, F108 and F127 demonstrate greater hydrophobicity compared to F68, which is reflected in the formulations’ elastic behavior. However, low-molecular-mass HA (10 and 200 kDa) can influence the hydrogels’ elasticity, particularly in the F127/NaCl formulation (Figure S2C). F108/MgCl_2_ and F127/MgCl_2_ are relatively stable and exhibit increased elasticity under frequency variation in the presence of high-molecular-mass HA (Figure S2E,F).

Complex viscosity changes against frequency revealed dilatant behavior during oscillation, particularly for comparatively more hydrophobic PL (F108 and F127) and associations of F68/MgCl_2_ with 200 and 1500 kDa HA. The viscosity of the F68/NaCl association remains relatively low and stable during oscillation (Figure S3A). Conversely, F68/MgCl_2_ exhibited nearly a 6-fold increase in viscosity at low frequency with 200 and 1500 kDa HA, compared to formulations with no HA or with 10 kDa HA (Figure S3D). F108/NaCl shows minimal differences between HA and non-HA types, maintaining a viscosity between 6 and 8 Pa s at low frequencies (Figure S3B).

The presence of MgCl_2_ in F108 formulations significantly alters the material resistance as the HA molecular mass increases (Figure S3E). The F108/MgCl_2_ system (without HA) exhibited a viscosity of approximately 5 Pa s. Upon incorporation of HA, a progressive increase in viscosity was observed: ∼7 Pa s for F108/MgCl_2_, ∼7.5 Pa s for F108/MgCl_2_, and ∼13 Pa s with 10, 200, and 1500 kDa of HA. In comparison, F127/NaCl systems—either without HA or with 10 kDa HA—displayed viscosity values like those of their F108/NaCl counterparts, maintaining values around 5 Pa s. However, with 200 and 1500 kDa HA, F127/NaCl tends to enhance its structural organization and resistance, exhibiting a viscosity between 11 and 13 Pa s (Figure S3C). In F127/MgCl_2_, a more structured hydrogel with higher viscosity is observed, especially when 200 kDa of HA is added, promoting the highest viscosity at low frequency among all formulations (Figure S3F).

The investigation of the materials’ behavior under intra-articular shear conditions is essential to discuss how frequency and dilution processes can affect the drug release to the target.^30^ Madkhali and co-workers pointed out that the synovial fluid viscosity and storage and loss moduli are altered in regular articular movements, like as in knee joint walking frequency (0.5 Hz) with the *G*″/*G*′ ratio ranging between 0.6 and 2.2 and *G*″/*G*′ ranging between 0.5 and 3 under knee joint running frequency (2.5 Hz).^24^ Although the *G*″/*G*′ ratios of the investigated hydrogels do not coincide with those of the native synovial fluid, their viscoelastic and shear-thinning behavior under joint-like frequencies suggests potential suitability for intra-articular drug delivery, pending further optimization to better mimic the rheology of synovial environments. That is a significant result since our hydrogels can be promising viscosupplementation strategies in osteoarthritic joints, considering the degraded synovial fluid.^25,26^

The material’s response, when subjected to mechanical oscillation, depends on the level of interaction between the polymers and other components of the system. The gel strength parameter *S* is obtained by fitting using the following power law model (eq 5):

$$G\prime =S\times f^{\nu }$$ 5

where *f* is frequency and ν is a dimensionless unit. The parameter *S* is related to the dependence between the elastic modulus and frequency. As hydrophobicity is enhanced, the hydrogel becomes more elastic, rigid, and resistant (Table S1).

Hydrogels containing NaCl responded with increased gel strength in the presence of different HA molecular masses (Figure 3). F127 hydrogels with HA (200 kDa) have approximately five times the *S* value of the hydrogel without HA and approximately 2.5 times the value in those containing 10 and 1500 kDa. The pattern is similar in F108/NaCl hydrogels, although the *S* values are lower in the presence of 10 and 200 kDa HA. The behavior of the hydrogels is different when MgCl_2_ is added: F127/1500 kDa HA presents greater stiffness, almost twice that of the control, while in the presence of 10 kDa, it presents *S* similar stiffness to the control formulation and even decreases by two times in the presence of 200 kDa HA (Figure 3B).

**Figure 3:**
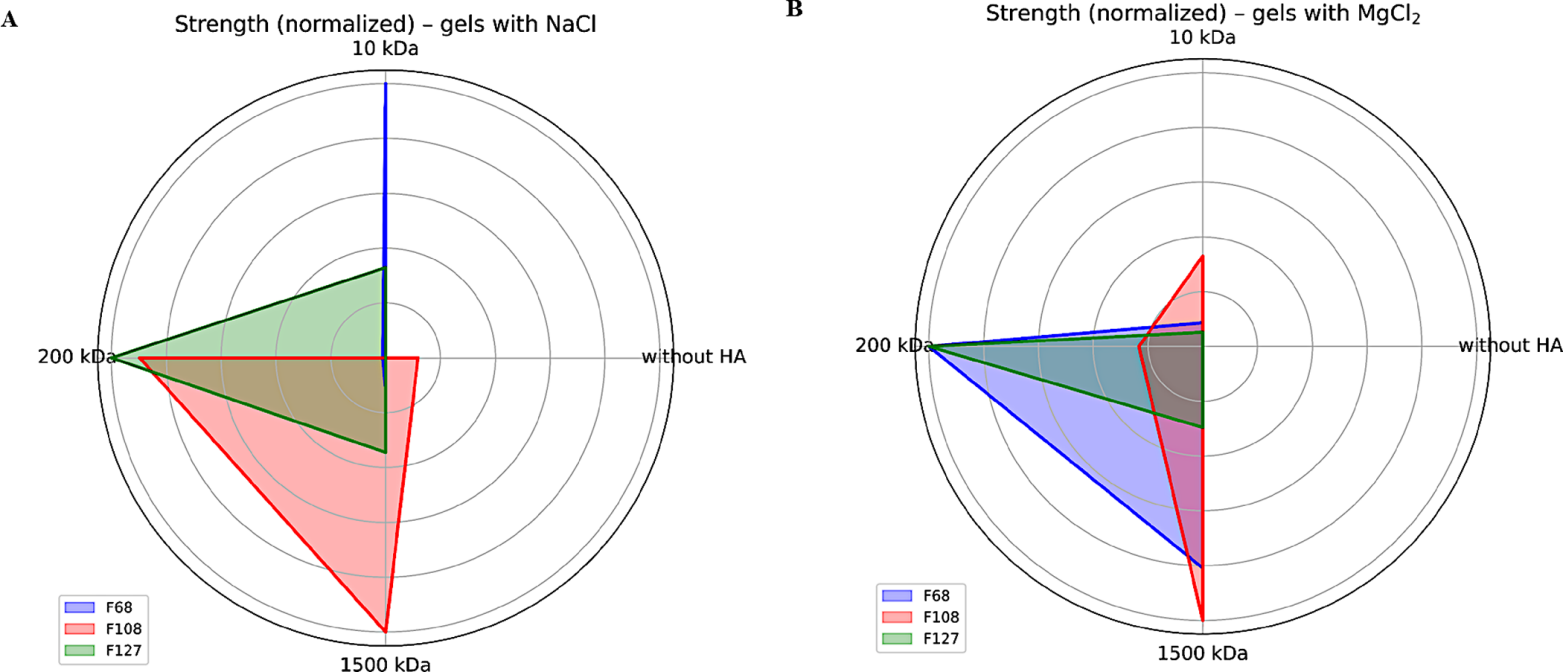
Normalized gel strength of F68, F108, and F127 20% (w/v) without hyaluronic acid (HA) or with 10, 200, and 1500 kDa HA 1% (w/v), with NaCl (154 mM) (A) and MgCl_2_ (0.9 mM) (B), at 37 °C. It is shown that F108 and F127 have higher strength with 200 kDa HA.

Compared to the control gel, no significant changes are observed in the F108/MgCl_2_ formulations. In the presence of MgCl_2_, the gel strength of the F68 hydrogels was strongly influenced by the molecular weight of hyaluronic acid. The incorporation of 200 kDa HA markedly increased the gel strength compared to the control and low-molecular-weight HA (10 kDa), indicating more efficient physical entanglement and reinforcement of the micellar network. However, when 1500 kDa HA was added, a slight decrease in strength was observed, although values remained substantially higher than those of the control formulation. This behavior suggests that excessively long HA chains may hinder optimal micellar packing due to steric effects, partially reducing network compactness despite stronger polymer–polymer interactions. These results show that a more hydrophobic PL tends to form more structured hydrogels by increasing micellar interactions. The molecular weight of the poloxamer influences that. F108 and F127, which have higher molecular weights (14,600 and 12,600 g/mol, respectively) compared to F68 (8400 g/mol), exhibited greater structural integrity over time. This behavior can be attributed to the formation of larger micelles and more extensive intermicellar entanglements, which enhance the network’s resistance to disruption. Conversely, F68, with shorter block lengths and higher hydrophilicity, forms smaller micelles and weaker interconnections, resulting in lower gel stability under equivalent conditions. The addition of salts also causes stronger entanglements due to the expulsion of water molecules from inside the structures, especially in the presence of MgCl_2_. The HA plays different roles depending on its molecular mass: 10 kDa HA is too small to impact the micelle–micelle interaction; on the contrary, 1500 kDa HA can inhibit this interaction due to its longer entanglements, but 200 kDa HA promotes higher strength and viscosity by facilitating the interaction like a hydrophilic “linker” between PEO chains of different micelles.^5^

### 3.3 Step 3: Material Resistance and Injectability

To understand the effects of HA on material structuring and stability and ultimately its applicability, flow curves, amplitude sweep measurements, and injectability studies were conducted. Viscosity decreases as the shear rate increases, displaying a typical pseudoplastic (shear-thinning) behavior (Figure 4). This shear-thinning response indicates structural breakdown and alignment of micelles and polymer chains under flow, which is characteristic of pseudoplastic hydrogels such as those based on PLs and HA. In formulations containing NaCl, the viscosity at low rates tends to be higher relative to the HA molecular mass and the type of PL. Additionally, the area between the forward and backward curves (or hysteresis) is approximately similar, regardless of the HA type. The presence of hysteresis indicates that there is a separation between the PL and HA chains as the shear rate increases (forward curve). Furthermore, when the shear rate is decreased (backward curve), the viscosity does not fully recover to the initial values, indicating that the formulations exhibit low structural recovery or low thixotropic memory. When considering an intra-articular application, this condition may be ideal for use, as articular movements provoke disaggregation of the formulation, leading to drug release. However, in the presence of MgCl_2_, the formulations show increased resistance in both curves and lower hysteresis than NaCl formulations, which may compromise ease of application, promote hydrogel rigidity, and diminish drug availability.

**Figure 4:**
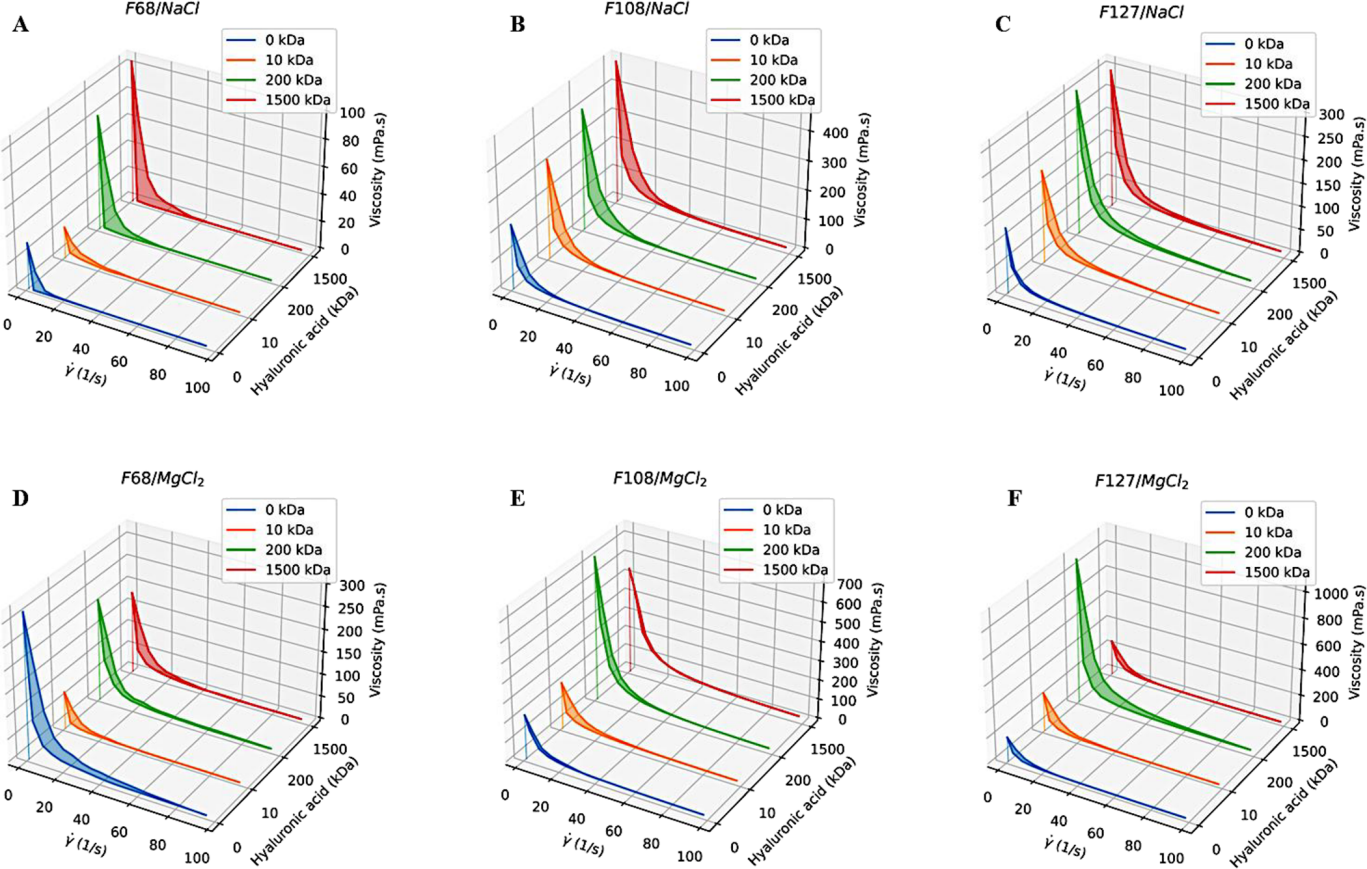
Flow curves (viscosity η as a function of shear rate γ̇) of 20% (w/v) F68/NaCl (A), F108/NaCl (B), F127/NaCl (C), F68/MgCl_2_ (D), F108/MgCl_2_ (E), and F127/MgCl_2_ (F), without hyaluronic acid (HA) or with 1% (w/v) 10, 200, and 1500 kDa HA, at 37 °C. The viscosity decreases as γ̇ increases, indicating dilatant hydrogels.

Additionally, the difference between forward and backward curves (hysteresis) describes how stable the material is under shear. F108/MgCl_2_ and F127/MgCl_2_ formulations show lower hysteresis than those with NaCl, and consequently, it is harder to disaggregate inside articulation.

To analyze the effect of the shear rate on viscosity and the formation/rupture of linkages,^27^ the Cross model (eq 6) was used:

$$\eta (\mathrm{\gamma ̇})=\eta_{\infty }+\frac{\eta_{0}-\eta_{\infty }}{1+(\tau \times \mathrm{\gamma ̇}{)}^{m}}$$ 6

where η_∞_ is viscosity in infinity, η_0_ is viscosity at γ̇ = 0, τ is relaxation time (or rupture of linkages), and *m* is a viscosity index.

The addition of HA to NaCl-containing PL systems induces distinct and complex effects on their rheological properties, particularly η_0_ and τ. These effects vary depending on HA molecular mass, the type of Pluronic used (F68, F108, or F127), and the direction of the measurement (forward vs backward curves), highlighting the dynamic nature of these polymer–salt–HA interactions.

In the F68/NaCl system, the addition of HA led to a significant increase in η_0_ in the forward direction, with values rising by over 100% compared to the formulation without HA (Table 2 and Table S2). Interestingly, this increase in viscosity was followed by a reduction in τ, indicating faster relaxation despite the thickening of the solution. This behavior suggests that although HA contributes to an increase in viscosity, it may not significantly enhance the elastic character of the system.

**Table 2:** Mean Difference Δη_0_ and Δτ from Forward and Backward Curves, Comparing F68/NaCl, F108/NaCl, and F127/NaCl with No HA (Control) and Those Containing 10, 200, and 1500 kDa HA, at 37 °C

System	Mean Δη_0_ Forward	Mean Δη_0_ Backward	Mean Δτ Forward	Mean Δτ Backward
F68/NaCl	+114.2%		–33.3%	
F108/NaCl	+87.7%	+17.4%	0.0%	–34.4%
F127/NaCl	–61.6%	–0.4%	+61.6%	–90.0%

The F108/NaCl system exhibited a more balanced response to HA. Here, η_0_ increased moderately in both directions, more prominently in the forward direction (+88%). While τ remained relatively unchanged in the forward curves, it decreased backward (−34%). This indicates that HA increases the overall viscosity, but the structure relaxes more quickly under shear reversal. Such a pattern suggests the formation of moderately robust structures that are partially disrupted under changing flow conditions (Table 2 and Table S2).

In contrast, the F127/NaCl system showed the most asymmetric behavior. The addition of HA led to a significant decrease in η_0_ in the forward direction, while τ increased markedly, suggesting the emergence of elastic structures. However, in the backward direction, τ decreased sharply by up to 90% (Table 2), indicating a near-complete loss of these structures under shear reversal. This suggests that F127/NaCl forms shear-sensitive networks that are built under flow but rapidly disintegrate when the direction changes.

These effects also varied according to HA molecular mass. 10 kDa HA typically produced the strongest shifts in viscosity and relaxation time, while higher mass (200 and 1500 kDa) showed more nuanced, system-dependent responses (Table S2). These findings underscore the nonlinear and material-specific interplay between HA and PL–salt matrices. Adding HA to NaCl-containing PL solutions leads to strikingly different rheological behaviors, depending on the PL used. These differences reflect not only the intrinsic properties of the polymers but also how HA influences micelle organization and system dynamics.

In the F68/NaCl system, HA causes a strong increase in viscosity (η_0_), particularly with a higher HA molecular mass. This suggests that HA, likely through excluded volume effects and crowding, enhances the effective resistance to flow. Interestingly, despite the increase in viscosity, the relaxation time (τ) decreases. This indicates a system in which structural rearrangement becomes faster, likely due to HA disrupting or fluidizing the micellar interactions rather than reinforcing them (Figure 5C).

**Figure 5:**
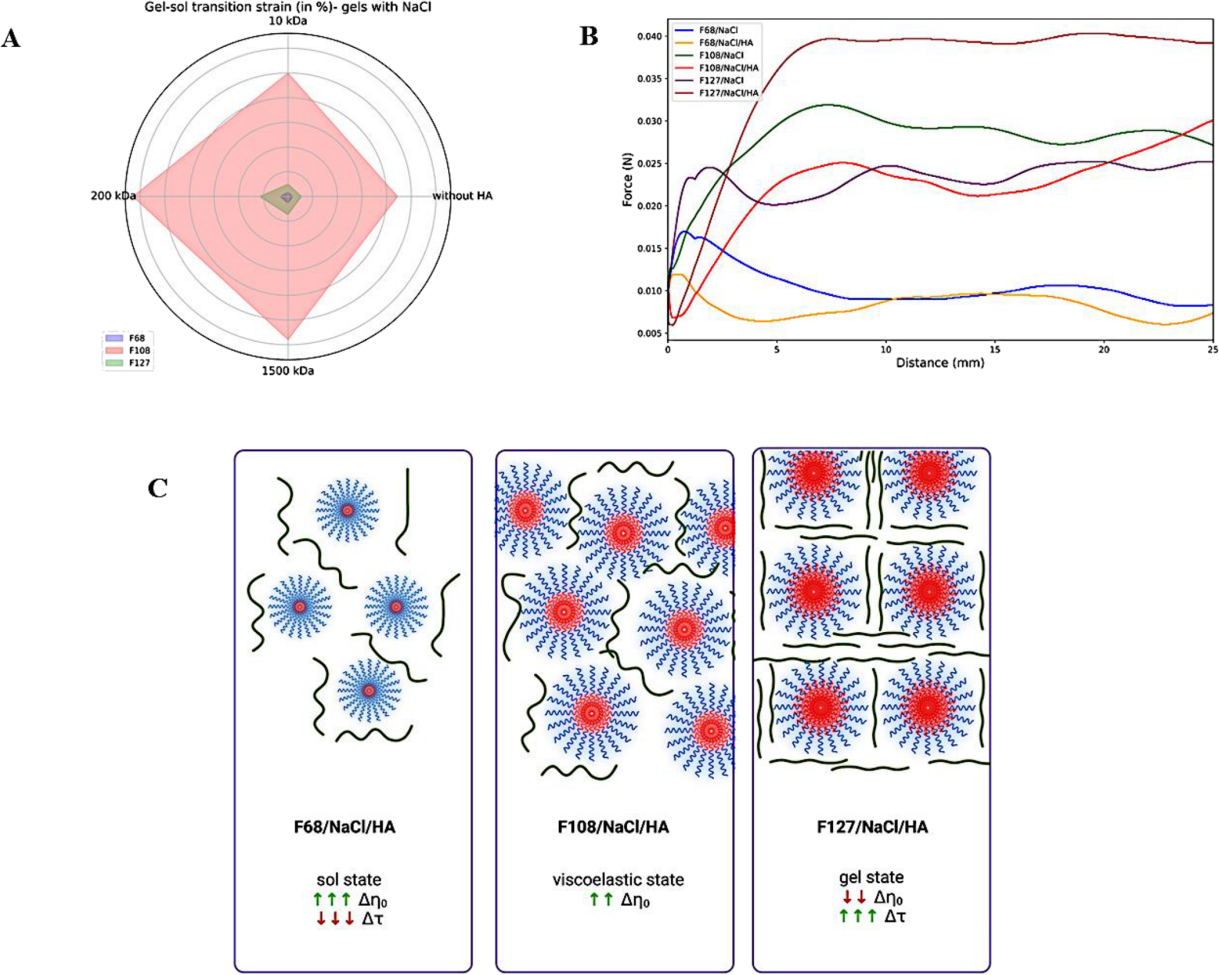
(A) Radar plot of gel–sol transition strain (in %), where the hydrogel is disaggregated and turns from a gel state (*G*′ > *G*″) to a sol state (*G*′ < *G*″), of 20% (w/v) F68, F108, and F127, with NaCl (154 mM), without hyaluronic acid (HA) or with 1% (w/v) 10, 200, and 1500 kDa HA, at 37 °C. F108/200 kDa HA presented the greatest shear strain and resistance. (B) Texture analysis of F68, F108, and F127 hydrogels containing NaCl (154 mM), without HA or with 200 kDa, at 25 °C. It is seen that the type of PL affects material resistance. The presence of HA enhances mechanical response of the material, demanding higher force and work to inject. (C) Schema of the molecular configuration of F68, F108, and F127 micelles in the presence of 200 kDa HA chains (green lines), at 37 °C. Compared to the formulation without HA, the F68/HA hydrogel is in a sol state but presenting elevated viscosity variation (Δη_0_) and decreased relaxation time variation (Δτ). F108/HA displays less elevated Δη_0_, a constant Δτ, and a moderated structural organization. F127/HA presents a well-organized, compacted structure yet with lower Δη_0_ and much higher Δτ than F127.

By contrast, the F108/NaCl system exhibits a more balanced behavior. HA consistently increases viscosity, while the relaxation time remains stable or increases slightly. This indicates a more elastic or structured fluid, where HA strengthens micelle–matrix interactions, possibly by forming transient bridges or participating in hydration shell modifications. These changes maintain fluid integrity while enhancing mechanical resistance (Figure 5C).

In the F127/NaCl system, the behavior is reversed: adding HA results in a significant decrease in viscosity despite a marked increase in τ. This implies that HA is not thickening the system but is modifying the micellar architecture, possibly by interfering with the tight micelle packing that F127 forms. The outcome is a less viscous yet more elastic and slow-relaxing material, which may reflect internal structuring or polymer entanglement that does not directly translate to flow resistance (Figure 5C). These diverse responses reveal the nuanced interplay between the micelle size, structure, ionic environment, and HA molecular weight.^5,28,29^

HA alters the rheological behavior of NaCl–PL systems in diverse ways. F127/NaCl appears highly tunable but shear-sensitive, ideal for applications where flow-triggered release or responsiveness is desired. F68/NaCl provides viscosity enhancement without significant changes in elasticity, and F108/NaCl offers a balance of both. These insights can guide the design of HA-enhanced soft materials with tailored mechanical properties for biomedical or industrial applications.^2,3,30^

The interaction between PL/HA chains enhances hydrogel strength under shear strain variation as the molecular weight of HA chains increases (Figure 5 and Table S1), mainly in the presence of 200 kDa HA (Figure 5A). F108/NaCl/200 kDa HA displays the most significant resistance to shear strain (γ = 126%), compared to F68/NaCl/200 kDa HA (γ = 3.5%) and 127/NaCl/200 kDa HA (γ = 23%). It reveals that higher strain is necessary to promote the gel–sol transition, i.e., to disorganize the hydrogel. Thus, F68 systems tend to be more shear-sensitive than the other two formulations. On the other hand, F108 systems demand higher shear to transit from gel to sol state. F127 systems present an intermediate pattern, which can facilitate the development of a hydrogel that shows good mechanical properties for intra-articular application.^31^

Although F68, F108, and F127, associated with NaCl and 200 kDa HA, demonstrate adequate sol–gel transition temperature, viscosity, and mechanical stability, these formulations must also demonstrate easy injectability. They demand a similar injection force (0.015–0.040 N) (Figure 5B). Nevertheless, the work required to expel the hydrogel from a syringe depends on the type of PL and the addition of HA, but just the comparison among PL types is significant (*p* < 0.05) (Table 3). It is seen that only F68/NaCl with 200 kDa HA (0.26 N mm) requires less work than F68/NaCl (0.36 N mm). On the contrary, F108/NaCl and F127/NaCl with 200 kDa need higher injection work than systems without HA, but it is still within acceptable limits of injectability.^21,32,33^ These formulations containing NaCl and 200 kDa do not present major mechanical differences. Furthermore, their structural stability, relatively high gel–sol transition, and relatively low injection work show the possibility of intra-articular application.

**Table 3:** Mechanical and Structural Analyses of F68/NaCl, F108/NaCl, and F127/NaCl Formulations with or without 200 kDa Hyaluronic Acid (HA)^a^

System	*W* (N mm)	*D̅*_H_ **(nm)**	PDI	ζ (mV)
F68/NaCl	0.36 ± 0.01	58.1 ± 13.8	0.371 ± 0.143	6.3 ± 1.9
F108/NaCl	0.17 ± 0.03	24.6 ± 0.5	0.303 ± 0.005	31.9 ± 11.0
F127/NaCl	0.67 ± 0.09	21.6 ± 0.7	0.126 ± 0.052	16.0 ± 1.9
F68/NaCl/HA	0.26 ± 0.02	325.0 ± 52.3	0.324 ± 0.011	0.2 ± 0.1
F108/NaCl/HA	0.62 ± 0.07	62.4 ± 3.2	0.491 ± 0.025	6.6 ± 1.9
F127/NaCl/HA	0.82 ± 0.17	143.0 ± 0.6	0.731 ± 0.009	7.1 ± 1.9

a Injection work, *W*, at 25 °C, *Z*-average hydrodynamic diameter (*D̅*_H_), polydispersity distribution index (PDI), and zeta potential (ζ) measurements were performed at 37 °C (*N* = 3).

### 3.4 Step 4: Micelle Stability

The stability of the micelles might be related to their ability to self-assemble at physiological temperature, as evidenced by measuring the hydrodynamical diameter and avoiding micellar aggregation through potential zeta measurement. At 37 °C, F68/NaCl micelles are not completely formed since its critical micelle temperature (CMT) is around 40 °C, so in the solution, there are some PL unimers (around 5 nm) and some micelles with 76 and 274 nm (Figure 6), highlighting some aggregation (Table 3 and Figure 6). Adding 200 kDa to F68/NaCl promotes a decrease in the number of unimers in solution. Incorporation of HA slightly broadens the size distribution and increases the *Z*-average *D̅*_H_ from 58 to 325 nm, mainly due to a small population of larger aggregates, although the dominant micellar population remains below 300 nm. F108/NaCl and F127/NaCl form quite stable micelles at 37 °C, with *D̅*_H_ of 24.6 and 21.6 nm, respectively, and relatively small polydispersity distribution index (PDI) (0.303 and 0.126, respectively) (Figure 6). However, the presence of 200 kDa HA provoked an enhanced micellar diameter to 62.4 nm (in F108/NaCl/HA) and 143 nm (in F127/NaCl/HA) and higher PDI (Table 3). That indicates that HA chains affect how F108/NaCl and F127/NaCl behave.

**Figure 6:**
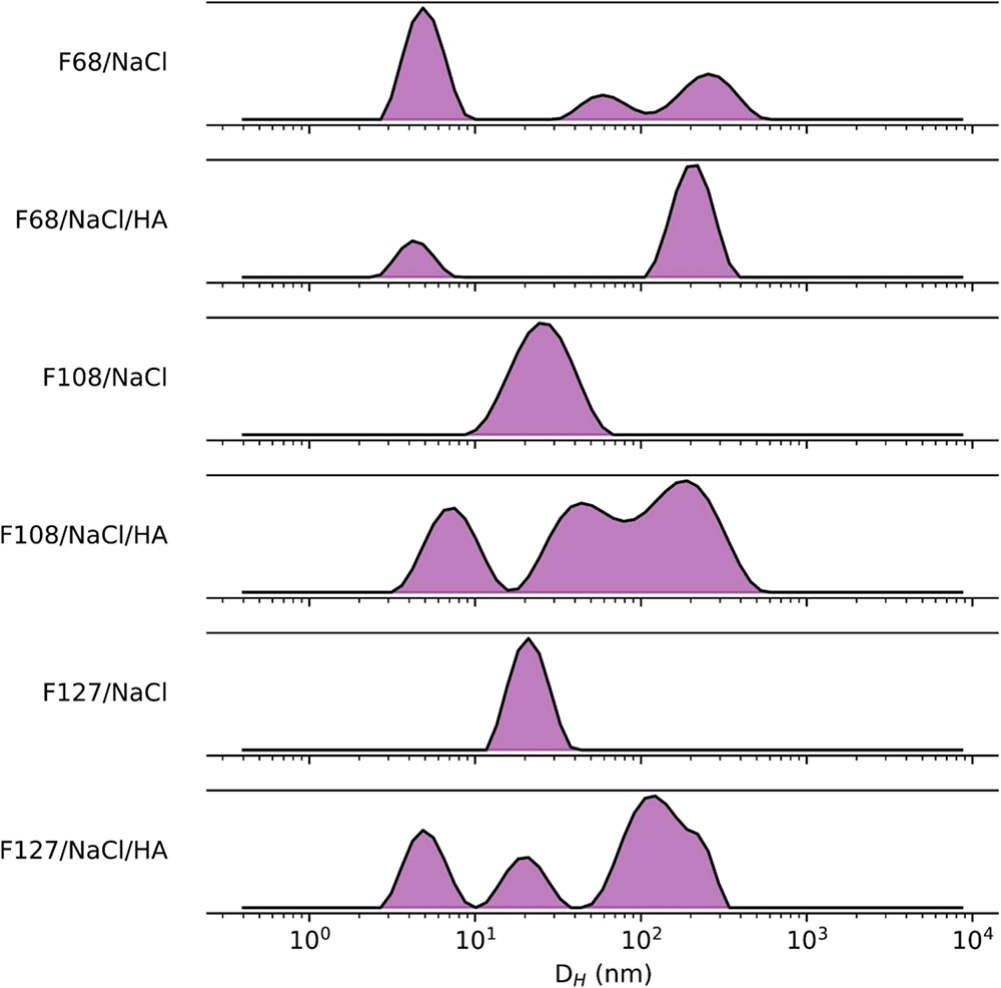
Distribution profile of the hydrodynamic diameter (*D*_H_), in nm, of 10% (w/v) F68, 5% (w/v) F108, and 5% (w/v) F127, without hyaluronic acid (HA) or with 1% (w/v) 200 kDa HA, at 37 °C. At this temperature, F68 chains do not group completely in micelles. F108 and F127 tend to form well-structured micelles of about 25 nm. In the presence of HA, agglutinations are also observed around 100 nm.

HA chains are expected to cause augmented micelle dimensions by forming an HA scaffold around the micelle with a hydrophilic interaction between HA and PEO chains. Zeta potential results show decreasing values when adding HA (Table 3). HA might also cause agglutination, as observed through a higher PDI and low negative charge in HA systems. Yet, it possibly cannot affect the incorporation of some drug into the micelle.^2,5,34^

### 3.5 Step 5: Drug Release Modulation

To study the formulation performance as drug delivery systems, release assays were carried out incorporating lidocaine (LDC), as a drug model. LDC is a widely used local anesthetic and antiarrhythmic drug with well-established safety and efficacy profiles. It is known as 2-(diethylamino)-*N*-(2,6-dimethylphenyl)­acetamide, with the molecular formula C_14_H_22_N_2_O and a molecular weight of approximately 234.34 g/mol. It appears as a white crystalline powder, readily soluble in water and alcohol, making it suitable for various pharmaceutical formulations, including micelles, liposomes, and hydrogels.^35–39^

The choice of the PL hydrogel formulation composition influences the drug release profile (Figure 7). In the absence of 200 kDa HA, the more complex PL matrix prevents the free diffusion of LDC outside the system. F68/NaCl and F127/NaCl hydrogels control nearly 25% (4.5 mg/mL) of LDC for up to 24 h in the experiment, whereas F108/NaCl released only 20% (4.0 mg/mL) (Figure 7A). The incorporation of HA 200 kDa progressively reduced the LDC release to ∼18% in the F68/NaCl/HA and F127/NaCl/HA formulations (3.5 mg/mL) and about 14% (2.5 mg/mL) in the F108/NaCl/HA formulation (Figure 7B).

**Figure 7:**
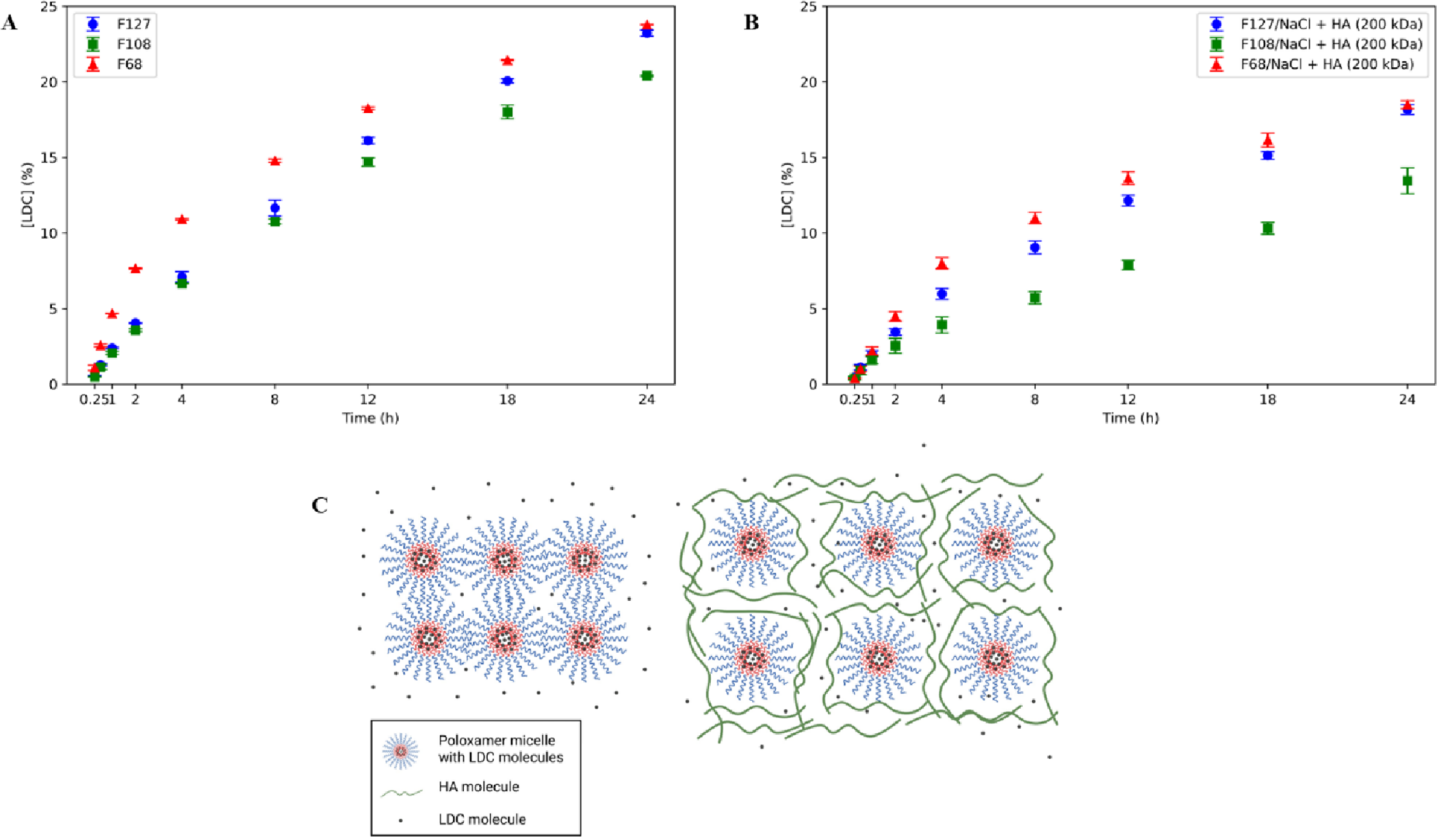
Lidocaine (LDC) release profiles of 20% (w/v) F68, F108, and F127 formulations, with NaCl (154 mM), (A) without hyaluronic acid (HA) or (B) with 1% (w/v) 200 kDa HA. HA chains hinder free diffusion of LDC by forming a polymeric matrix or by ionic interaction between LDC and the negative charge of HA carboxyl groups (C).

Aiming to characterize the drug release rate, mathematical models described in Section 2.4 were used. For all formulations, the Higuchi model provided the best fit (*R* ^2^ = 0.99), indicating that diffusion is the predominant release mechanism (Table 4). The Korsmeyer–Peppas model also provided a good correlation with diffusional exponent values (*n*) ranging from 0.64 to 0.82. These values fall within the anomalous (non-Fickian) transport range, suggesting a combination of Fickian diffusion and matrix relaxation (erosion or swelling).

**Table 4:** Release Constants Calculated from Zero-Order, Higuchi, Hixson–Crowell, and Korsmeyer–Peppas Models over Drug Release Profiles of F68/NaCl, F108/NaCl, and F127/NaCl with or without 200 kDa Hyaluronic Acid (HA), at 37 °C^a^

	Zero-order		Higuchi		Hixson–Crowell		Korsmeyer–Peppas		
	*K*_0_ (μg mL^–1^ h^–1^)	*R* ^2^	*K*_H_ (μg mL^–1^ h^–1^)	***R*** ^2^	*K*_HC_ (μg mL^–1^ h^–1^)	***R*** ^2^	*K*_KP_ (log μg mL^–1^ h^–1^)	*n*	*R* ^2^
F68/NaCl	184.8 ± 22.6	0.91	1036.0 ± 38.9	0.99	0.38 ± 0.08	0.74	1.42 ± 0.04	0.64 ± 0.05	0.96
F108/NaCl	173.2 ± 14.4	0.95	948.7 ± 22.8	0.99	0.44 ± 0.08	0.82	1.73 ± 0.03	0.81 ± 0.03	0.99
F127/NaCl	195.4 ± 14.1	0.96	1064.0 ± 24.9	0.99	0.46 ± 0.08	0.83	1.68 ± 0.03	0.81 ± 0.03	0.99
F68/NaCl/HA	158.0 ± 17.0	0.92	878.5 ± 25.0	0.99	0.42 ± 0.09	0.76	1.74 ± 0.05	0.82 ± 0.06	0.97
F108/NaCl/HA	108.0 ± 5.1	0.98	579.1 ± 26.8	0.99	0.35 ± 0.05	0.86	1.86 ± 0.02	0.72 ± 0.03	0.99
F127/NaCl/HA	162.5 ± 12.3	0.96	887.3 ± 15.9	0.99	0.43 ± 0.08	0.82	1.73 ± 0.03	0.79 ± 0.04	0.99

a *n* = 3/formulation.

In systems without HA, F127/NaCl exhibited the highest release rate constants (*K*_0_ = 195.4 μg mL^–1^ h^–1^ and *K*_H_ = 1064.0 μg mL^–1^ h^–1^). At the same time, F68/NaCl had the lowest, suggesting that the HLB and structural differences between poloxamers impact drug diffusion (Table 4). F127, being more hydrophobic and forming tighter micellar networks, appears to provide a matrix that facilitates lidocaine diffusion that is more robust than F68.

The incorporation of HA generally reduced the release rate constants across all models. For example, *K*_0_ for F108 decreased from 173.2 to 108.0 μg mL^–1^ h^–1^ with HA, and *K*_H_ decreased from 948.7 to 579.1 μg mL^–1^ h^–1^. This reduction likely results from an increased matrix viscosity and possible interaction between HA and PL micelles, which hinders LDC mobility. The slight changes in diffusional exponent (*n*) values, particularly an increase in the F68/NaCl/HA concentration (from 0.64 to 0.82), suggest that matrix relaxation becomes more influential in the presence of HA.

Among the HA-containing systems, F108/NaCl/HA demonstrated the lowest release rates, making it potentially suitable for sustained drug delivery applications (Figure 7C). The sustained release observed in these systems may be beneficial for reducing the dosing frequency and maintaining therapeutic concentration over time. HA chains tend to retard the free diffusion of LDC molecules. The diffusion of the LDC molecule is reduced by the entangled network formed by both HA and PL. Moreover, due to the highly hydrophilic environment promoted by HA, the water might decrease the mobility of a solute. Although LDC is a weak base and largely neutral at physiological pH, it can interact with the negative charges of carboxyl groups on the HA chain by hydrogen bonding and transient ionic interactions.^3,5,30,31,40,41^

## 4 Conclusions

Drug delivery systems present enormous potential for developing new therapeutic strategies. In this study, we propose five steps to simplify the study of drug carrier materials using rheological tools, texture, light scattering, and kinetic assays. This study demonstrates that the rheological behavior of poloxamer-based systems in the presence of hyaluronic acid (HA) and physiological salt concentrations is highly dependent on the PL type and HA molecular weight. While all systems exhibit some sensitivity to HA addition, their responses diverge markedly in viscosity, relaxation dynamics, and structural behavior.

HA increased the zero-shear viscosity in F68/NaCl solutions, especially with HA of high molecular mass, while accelerating structural relaxation. This indicates a fluid network dominated by crowding and excluded volume effects instead of stable associative interactions. F108/NaCl systems show a consistent and moderate increase in viscosity with minimal impact on the relaxation time, indicating a balanced interplay between HA and the micellar matrix, likely through soft structuring or weak interactions that enhance mechanical resistance without compromising flow dynamics.

F127/NaCl formulations show an opposing trend since HA leads to a substantial reduction in viscosity but a strong increase in the relaxation time. This suggests that HA may disrupt the dense micelle packing characteristic of F127 gels, thereby disturbing the network and promoting an elastic or entangled behavior. These effects are further modulated by HA molecular mass with high-kDa HA amplifying the observed trends.

At 20% (w/v) and physiological temperature (37 °C), each PL forms distinct physical states: F68 remains a micellar liquid; F108 exhibits viscoelastic structuring, and F127 transitions to a thermoresponsive gel. HA alters these states, which must be considered for biomedical applications, such as injectable gels, drug delivery vehicles, or tissue scaffolds. Together, these findings highlight the tunability of PL–HA systems and underscore the importance of polymer–biopolymer–salt interactions in designing advanced soft materials with tailored rheological profiles.

## Supplementary Material



## Notes

Viscosity and relaxation time calculated from the Cross model, gel strength, gel–sol transition shear strain, sol–gel transition temperature, *G*″/*G*′ frequency sweeps at 37 °C, viscosity frequency sweeps at 37 °C, flow curves at 37 °C, and amplitude sweeps at 37 °C (PDF)

## Abbreviations

CMC: critical micelle concentration
CMT: critical micelle temperature
EO: ethylene oxide
HA: hyaluronic acid
HLB: hydrophilic–lipophilic balance
LDC: lidocaine
PDI: polydispersity distribution index
PEO: poly­(ethylene oxide)
PL: poloxamer
PO: propylene oxide
PPO: poly­(propylene oxide)
