# Supplementary material for: Tuning Pluronic Hydrogel Properties via Ionic Strength and Hyaluronic Acid for Optimized Rheology and Drug Delivery Performances
Source: ACS Omega. 2025 Dec 4;10(49):60519–31. doi: 10.1021/acsomega.5c08070 (PMC12713464; doi:10.1021/acsomega.5c08070)
Supplement: Supplementary file 1 [file ao5c08070_si_001.pdf]

SUPPORTING INFORMATION

Tuning Pluronic Hydrogel Properties via Ionic Strength and Hyaluronic Acid for Optimized Rheology and Drug Delivery Performances

Anderson Ferreira Sepulveda[1,2*], Lucas Ferreira de Oliveira[1], Agatha Maria Pelosine[1], Wendel A. Alves[1], Daniele Ribeiro de Araujo[2*]

[1] Center for Natural and Human Sciences, Federal University of ABC, 09280-560, Santo André-SP, Brazil

[2] Department of Biophysics, Federal University of São Paulo, 04044-010, São Paulo-SP, Brazil

*Corresponding author: anderson.f.sepulveda@gmail.com; daniele.ribeiro@unifesp.br

Table S1: Parameters calculated from Cross model fitted over forward and backward curves (from flow curves) of 20 % (w/v) F68, F108, and F127, with 154 mM NaCl or 0.9 mM MgCl2, without hyaluronic acid (HA) – control - or with 1 % (w/v) 10 kDa, 200 kDa, and 1500 kDa HA, at 37 °C. (N = 3)

		Forward				Backward		
	**HA (kDA)**	η_0 **(Pa.s)**	η_∞ **(Pa.s)**	τ **(s)**	m	η_0 **(Pa.s)**	τ **(s)**	m
**F68/MgCl$_2$**	**Control**	332.7±4.6	3.6±1.5	0.13±0.01	1.9±0.1	157.9±25.4	0.21±0.04	1.6±0.1
	**10**	86.2±0.7	0.2±0.2	0.16±0.01	2.0±0.1	31.3±4.4	0.22±0.05	1.3±0.1
	**200**	229.7±2.3	6.4±0.6	0.13±0.01	2.4±0.1	124.8±12.9	0.15±0.02	2.3±0.2
	**1500**	189.4±1.1	1.1±0.3	0.14±0.01	2.0±0.1	120.6±13.4	0.28±0.04	1.3±0.1
**F68/NaCl**	**Control**	34.5±0.1	0.04±0.01	0.20±0.01	3.5±0.1	-	-	-
	**10**	25.4±0.8	~ 0	0.16±0.01	1.8±0.1	7.4±1.0	0.14±0.03	1.7±0.2
	**200**	86.7±0.2	~ 0	0.16±0.01	2.7±0.1	3.0±0.5	0.15±0.03	1.7±0.2
	**1500**	109.6±1	0.03±0.2	0.16±0.01	2.5±0.1	2.0±0.2	0.18±0.02	2.2±0.2
**F108/MgCl$_2$**	**Control**	380.7±35.5	5.41±1.07	0.13±0.01	1.7±0.1	245.8±4.1	0.33±0.04	1.3±0.1
	**10**	131.6±7.1	7.11±0.54	0.12±0.01	1.7±0.1	253.4±1.8	0.15±0.01	1.3±0.1
	**200**	953.2±80.4	10.6±1.2	0.13±0.01	2.0±0.1	782.0±4.6	0.28±0.03	1.5±0.1
	**1500**	834.0±74.1	22.8±2.3	0.12±0.01	2.0±0.1	573.4±7.9	0.21±0.03	1.5±0.1
**F108/NaCl**	**Control**	224.7±1.6	3.51±0.45	0.14±0.01	2.1±0.1	199.2±23.0	0.31±0.05	1.3±0.1
	**10**	345.5±0.7	4.98±0.22	0.13±0.01	2.1±0.1	170.4±8.4	0.17±0.01	1.5±0.1
	**200**	422.0±2.5	3.81±0.81	0.12±0.01	2.1±0.1	248.6±9.7	0.25±0.01	1.3±0.1
	**1500**	497.5±1.3	8.19±0.43	0.11±0.01	2.1±0.1	282.8±15.0	0.19±0.02	1.5±0.1
**F127/MgCl$_2$**	**Control**	193.0±1.0	1.62±0.30	0.14±0.01	1.9±0.1	324.2±47.8	0.64±0.11	1.2±0.1
	**10**	300.2±1.4	2.97±0.43	0.12±0.01	2.1±0.1	195.0±16.9	0.26±0.03	1.5±0.1
	**200**	1155±12	24.96±3.9	0.13±0.01	1.9±0.1	621.5±76.5	0.24±0.04	1.7±0.1
	**1500**	270.4±1.3	5.83±0.41	0.13±0.01	2.0±0.1	262.3±24.0	0.24±0.03	1.5±0.1
**F127/NaCl**	**Control**	836.5±36.0	0.69±0.13	0.47±0.02	1.2±0.1	246.5±4.7	2.63±0.14	1.1±0.1
	**10**	212.0±3.5	7.17±0.99	0.13±0.01	1.8±0.1	178.4±30.3	0.27±0.07	1.3±0.1
	**200**	440.3±64.3	9.35±1.26	0.12±0.01	1.9±0.1	326.0±4.4	0.32±0.06	1.5±0.1
	**1500**	312.4±3.6	8.14±0.95	0.13±0.01	2.1±0.1	232.0±24.1	0.20±0.03	1.7±0.1

Table S2: Gel strength S and power-law index ν, fitted over frequency sweeps at 37 °C. The fifth column shows the shear strain when $G' = G''$ obtained from amplitude sweeps at 37 °C.

	HA (kDa)	$S\ (Pa.s^{-\nu})$	ν	γ (%)
**F68/MgCl₂**	Control	434.2 ± 18.2	0.34 ± 0.03	5.8
	10	813.5 ± 38.6	0.43 ± 0.03	7.8
	200	4830.0 ± 100.5	0.22 ± 0.01	6.4
	1500	3991.0 ± 122.0	0.27 ± 0.02	6.3
**F68/NaCl**	Control	0.15 ± 0.04	2.45 ± 0.11	3.9
	10	9.91 ± 2.24	1.55 ± 0.11	3.5
	200	0.33 ± 0.04	2.10 ± 0.06	6.4
	1500	1.25 ± 0.15	1.47 ± 0.06	5.5
**F108/MgCl₂**	Control	6909.0 ± 48.6	0.07 ± 0.01	15.0
	10	8124.0 ± 57.7	0.06 ± 0.01	80.0
	200	7767.0 ± 19.9	0.03 ± 0.01	90.0
	1500	10587.0 ± 60.5	0.04 ± 0.01	90.0
**F108/NaCl**	Control	7323.0 ± 49.8	0.08 ± 0.01	89.0
	10	7012.0 ± 75.2	0.12 ± 0.01	100.0
	200	9375.0 ± 96.9	0.11 ± 0.01	126.0
	1500	9645.0 ± 120.0	0.10 ± 0.01	116.0
**F127/MgCl₂**	Control	7817.0 ± 74.9	0.09 ± 0.01	12.6
	10	8952.0 ± 270.4	0.23 ± 0.02	14.3
	200	29567.0 ± 271.8	0.05 ± 0.01	13.3
	1500	14252.0 ± 114.6	0.08 ± 0.01	10.3
**F127/NaCl**	Control	7312.0 ± 47.0	0.08 ± 0.01	10.9
	10	9642.0 ± 143.1	0.16 ± 0.01	10.0
	200	14388.0 ± 151.7	0.09 ± 0.01	23.0
	1500	9754.0 ± 71.8	0.10 ± 0.01	14.8

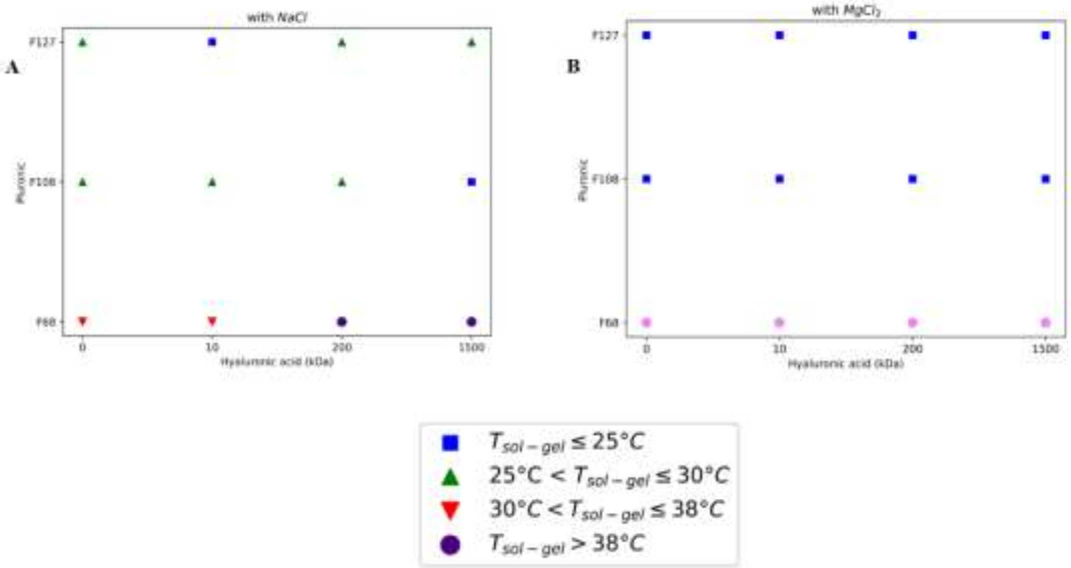

Figure S1: Sol-gel transition temperatures ($T_{sol\text{-}gel}$) of 20 % (w/v) F68, F108, and F127, without hyaluronic acid (HA) – 0 kDa – or with 1 % (w/v) 10 kDa, 200 kDa, and 1500 kDa, containing 154 mM NaCl (**A**) or 0.9 mM $MgCl_2$ (**B**). It is observed that the presence of higher molecular weight HA and/or $MgCl_2$ provokes lower transition temperature, indicating that they promote higher hydrogel structuring.

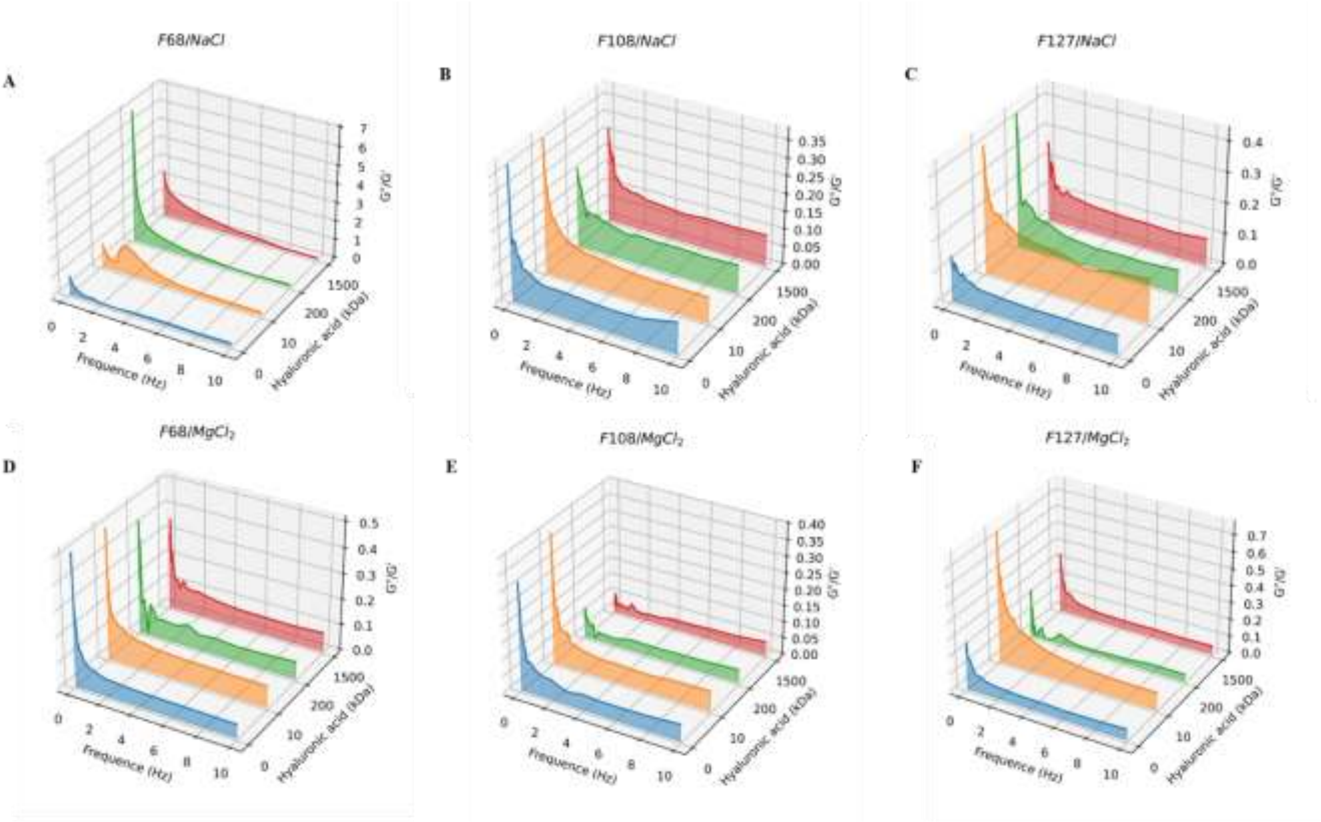

Figure S2: Frequency sweeps (G''/G' in function of frequency) at 37 °C of 20 % F68/NaCl (**A**), F108/NaCl (**B**), F127/NaCl (**C**), F68/$MgCl_2$ (**D**), F108/$MgCl_2$ (**E**), and F127/$MgCl_2$ (**F**), without hyaluronic acid (HA) and with 1 % (w/v) 10 kDa, 200 kDa, and 1500 kDa HA. As the frequency increases, the ratio G''/G' tends to diminish, pointing out that the mechanical oscillation promotes more elastic behavior of the hydrogel, mainly in the presence of NaCl.

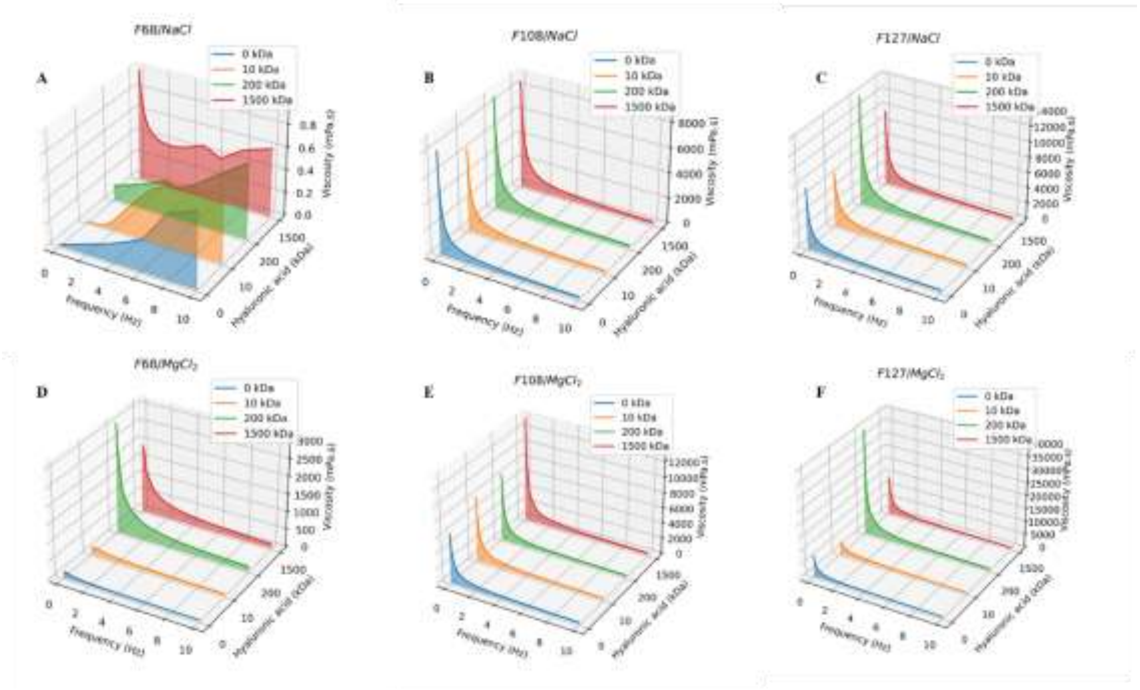

Figure S3: Frequency sweeps (viscosity in function of frequency) at 37 °C of 20 % F68/NaCl (**A**), F108/NaCl (**B**), F127/NaCl (**C**), F68/$MgCl_2$ (**D**), F108/$MgCl_2$ (**E**), and F127/$MgCl_2$ (**F**), without hyaluronic acid (HA) and with 1 % (w/v) 10 kDa, 200 kDa, and 1500 kDa HA. Except for the F68/NaCl formulations, the viscosity decreases as frequency increases, due to dilatant behavior. It does not happen in F68/NaCl formulations because of the sol state of F68 solutions, since it is below the sol-gel transition temperature.

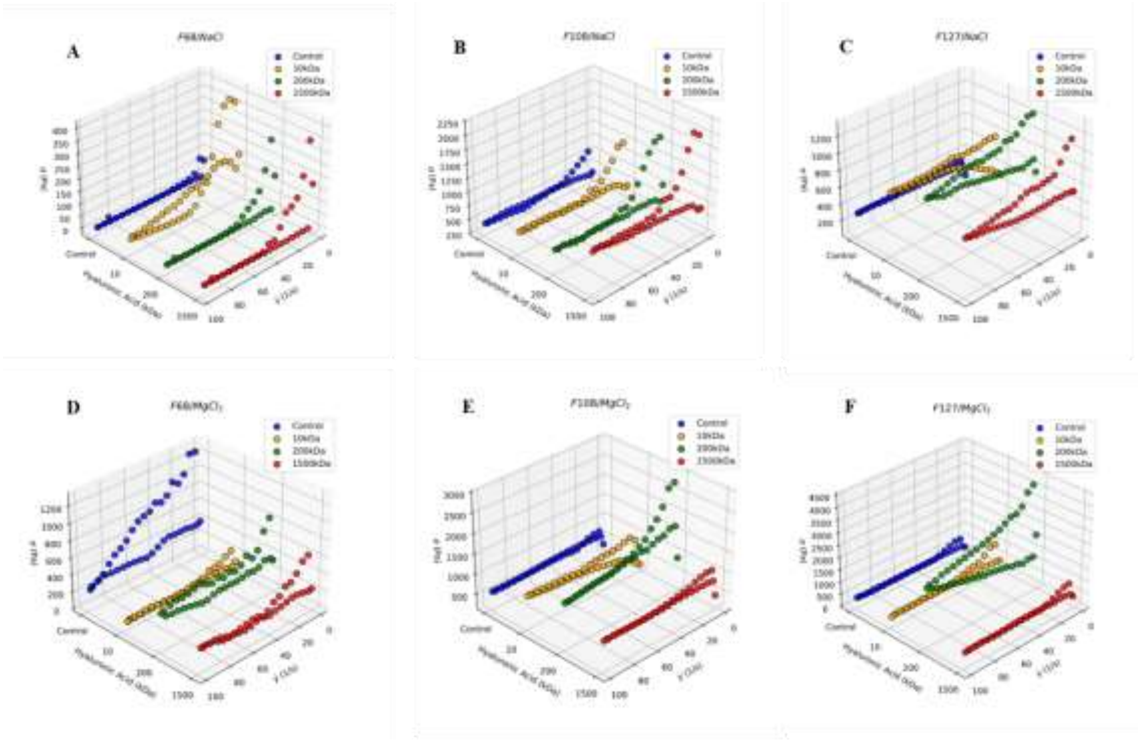

Figure S4: Flow curves (shear stress σ in function of shear rate $\dot{\gamma}$) at 37 °C of 20 % F68/NaCl (**A**), F108/NaCl (**B**), F127/NaCl (**C**), F68/$MgCl_2$ (**D**), F108/$MgCl_2$ (**E**), and F127/$MgCl_2$ (**F**), without hyaluronic acid (HA) and with 1 % (w/v) 10 kDa, 200 kDa, and 1500 kDa HA. It is observed that the presence of HA causes higher shear stress and an increasing difference between forward and backward curves, and consequently increased hysteresis, especially in F127 formulations, indicating that the presence of HA affects the structural organization of the F127 system by promoting disruption as shear rate increases. Moreover, these results show that $MgCl_2$ promotes a more resistant hydrogel than NaCl.

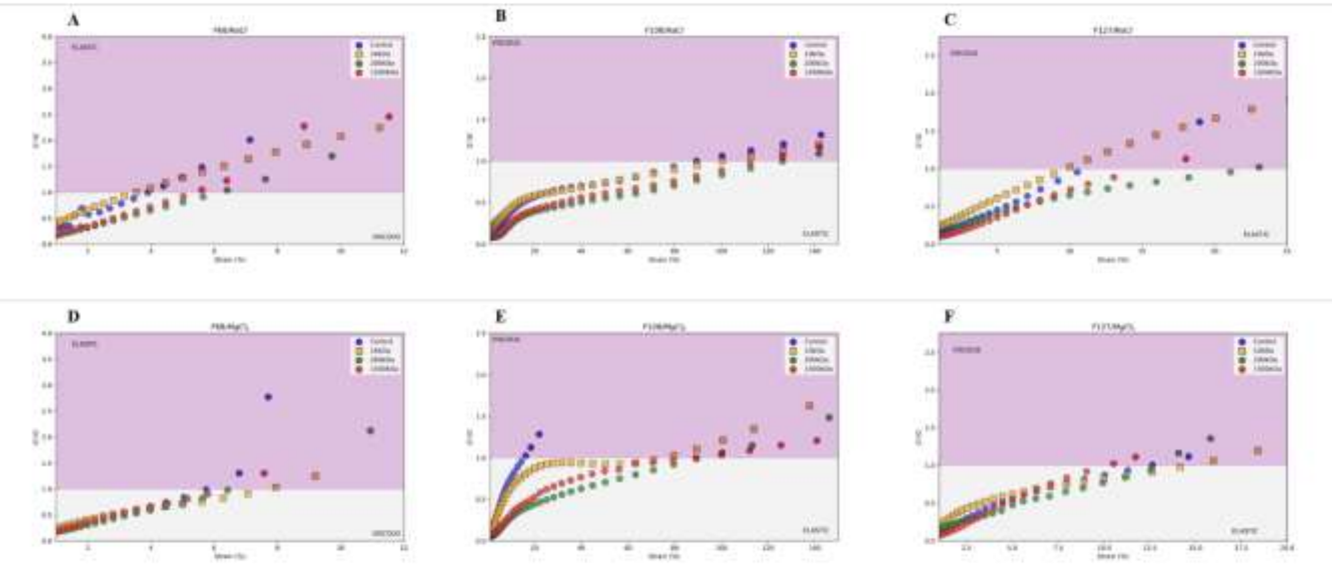

Figure S5: Amplitude sweeps (ratio G''/G' in function of shear strain γ at 37 °C of 20 % F68/NaCl (**A**), F108/NaCl (**B**), F127/NaCl (**C**), F68/$MgCl_2$ (**D**), F108/$MgCl_2$ (**E**), and F127/$MgCl_2$ (**F**), without hyaluronic acid (HA) and with 1 % (w/v) 10 kDa, 200 kDa, and 1500 kDa HA. As shear strain increases, the hydrogel tends to transform from elastic (G' > G'') to a viscous state (G' < G''), where a more disorganized structure predominates.
